# Design of a Multi-Mode Hybrid Micro-Gripper for Surface Mount Technology Component Assembly

**DOI:** 10.3390/mi14071464

**Published:** 2023-07-21

**Authors:** Gianmauro Fontana, Nicola Iacono, Simone Pio Negri, Gabriele Papadia

**Affiliations:** 1 Institute of Intelligent Industrial Technologies and Systems for Advanced Manufacturing CNR-STIIMA, Via A. Corti 12, 20133 Milan, Italy; 2 Department of Mechanical and Industrial Engineering, University of Brescia, Via Branze 38, 25123 Brescia, Italy; 3 Institute of Intelligent Industrial Technologies and Systems for Advanced Manufacturing CNR-STIIMA, Via P. Lembo 38/F, 70124 Bari, Italy; 4 Department of Innovation Engineering, University of Salento, Via per Monteroni, 73100 Lecce, Italy

**Keywords:** hybrid micro-gripper, vacuum micro-gripper, two-finger micro-gripper, SMT assembly, closed-loop control of stepper motor, PID controller, embedded system

## Abstract

In the last few decades, industrial sectors such as smart manufacturing and aerospace have rapidly developed, contributing to the increase in production of more complex electronic boards based on SMT (Surface Mount Technology). The assembly phases in manufacturing these electronic products require the availability of technological solutions able to deal with many heterogeneous products and components. The small batch production and pre-production are often executed manually or with semi-automated stations. The commercial automated machines currently available offer high performance, but they are highly rigid. Therefore, a great effort is needed to obtain machines and devices with improved reconfigurability and flexibility for minimizing the set-up time and processing the high heterogeneity of components. These high-level objectives can be achieved acting in different ways. Indeed, a work station can be seen as a set of devices able to interact and cooperate to perform a specific task. Therefore, the reconfigurability of a work station can be achieved through reconfigurable and flexible devices and their hardware and software integration and control For this reason, significant efforts should be focused on the conception and development of innovative devices to cope with the continuous downscaling and increasing variety of the products in this growing field. In this context, this paper presents the design and development of a multi-mode hybrid micro-gripper devoted to manipulate and assemble a wide range of micro- and meso-SMT components with different dimensions and proprieties. It exploits two different handling technologies: the vacuum and friction.

## 1. Introduction

In the last few decades, the rapid advancements in the field of electronic technologies have led to the development of enabling technologies, processes, and devices in many fields, including smart manufacturing, biomedical, and aerospace.

Nowadays, the Printed Circuit Boards (PCBs) of electronic systems can integrate few or hundreds of different Surface Mount Technology (SMT) components (e.g., resistors, capacitors, ball grid array packages, etc.) with dimensions ranging from the micro-scale to the meso-scale. Indeed, a heavy trend to miniaturization has emerged, manifesting the necessity of acquiring smaller electronic systems, integrating an increasing number of functionalities [1].

Moreover, the customization trend of electronic products requires manufacturing systems that can be rapidly reconfigured and optimized for wide varieties of production batches, even very small ones [2].

These trends limit the overall performance of current automated manufacturing systems and introduce new challenges related to demanding specifications to be addressed with enhanced or new manufacturing processes, innovative devices and tools, and advanced strategies. The automated manipulation and assembly of tiny-sized SMT components require high precision, reliability, throughput, flexibility, and low-cost solutions that are difficult to be achieved by exploiting the conventional systems [3]. Current PCB manufacturing systems are weak in these capabilities. SMT placement machines typically can represent the bottleneck of SMT assembly lines, becoming the key issue for assembly line optimization [4]. The assembly process involves placing the SMT components onto the PCB pads, that are then soldered onto the board. The placement precision required for SMT micro-components is typically on the order of 10–50 microns [5], which can be difficult to achieve with traditional pick-and-place machines. Furthermore, SMT components can be damaged from excessive heat during soldering, so careful temperature control is necessary during this operation.

High-throughput automated assembly work-cells, integrating high-performance robots, gripping tools, soldering devices, sensing systems, and control strategies, are required for new demanding PCBs, for example, for devices with embedded components in the inner layer of a rigid PCB [6], of flexible PCBs, or of solder-free PCBs, or 3D Molded Interconnect Device [7].

The increasing awareness of the need to manage different SMT components, often of tiny sizes, has stimulated the development of new approaches toward efficient and flexible technologies to enable PCB assembly. The development of flexible and reconfigurable tools, consisting of standardized modules and minimum application-specific elements, is a key approach to support and improve the adaptability to different automated processes while saving set-up costs [8].

Manufacturers and researchers are investigating and developing new PCB assembly machine architectures and manipulation methods to increase the automated work-cell performance and cope with the new demanding requirements.

In PCB assembly automation, sensors are utilized to gather specific information about the robot end-effector, the part to manipulate, and the working environment. Due to the small dimensions of components, the use of high-precise positioning equipment and fixturing can be extremely difficult; therefore, vision and force systems are crucial in automated micro-manipulation work-cells. These systems can address various issues, such as inspection, quality control, supervision, and vision-based robot control [9,10].

Vision-based control and robot guidance enhance work-cell flexibility and autonomy, exploiting visual information to implement strategies like look-and-move or visual servoing.

Monitoring the interaction between the manipulator and micro-part ensures the correct manipulation with the desired force and prevents damage to fragile micro-components. Force sensing methods can be employed for robot control, implementing hybrid position–force or impedance control strategies. Moreover, equipping the gripper with a force sensor allows for tactile control during tasks [11].

Due to the high precision demand, the calibration of sensing systems should ensure high measurement performance with high resolutions [12]. For this reason, new calibration techniques have been investigated and proposed [13,14].

Human–robot collaboration is a key research trend in the evolution of robot control, driven by Industry 4.0 [15]. This collaboration combines human adaptability with robot accuracy, enabling innovative and efficient production strategies. Collaborative robotics, with safety requirements recently outlined by [16], diverges from traditional robotics, characterized by heavy and strong machines operating in cages inaccessible to the operator. Collaborative robots are typically lightweight and intrinsically safe, greatly enhancing flexibility. This flexibility can play an important role in PCB manufacturing processes that require rapid and customized changes in the tasks. For example, a collaborative task where the robot manipulates the SMT components and the human operator solders them to the PCB substrate could be a smart approach in the small batch production of electronics products.

Furthermore, suitable micro-grippers are fundamental for the successful execution of the PCB assembly operations since the performance of the conventional gripping tools is limited by the influence of adhesion forces at the micro-scale (e.g., electrostatic, van der Waals, and capillary forces) and by the heterogeneity of the SMT components to manipulate that are characterized by different dimensions and properties (e.g., shape, material, and color) [17].

A new generation of flexible devices, such as grippers to be mounted on the end-effector of the robotic system, would foster the achievement of suitable automated systems. Indeed, many specific tools, capable of micron resolution, have to be available during the different phases of the assembly process, such as the soldering, gripping, and releasing of the components [18]. However, the need for a wide set of tools introduces several challenges, resulting in a more complex, expensive, and time-consuming process. The study and development of innovative flexible tools, able to optimize one or more phases of the assembly process, will overcome the above-mentioned current limits [19].

Several types of micro-grippers were designed for SMT assembly, including electrostatic grippers, capillary grippers, vacuum grippers, and friction grippers [20]. Electrostatic grippers use the attraction between charged plates to grip SMT components. They are small, lightweight, and can handle components as small as 50 µm. However, they require high voltages to operate and are susceptible to interference from external electric fields [21,22,23]. Capillary grippers use a small drop at the surface of the gripper in order to create a liquid bridge and the meniscus to increase this force between gripper and part [24,25,26,27,28]. Vacuum grippers use suction to grip SMT components. They are versatile, can handle fragile components of different sizes and shapes, and require low power. However, they can be easily occluded, and both the gripper and the part need to have smooth surfaces to prevent air leakage [29,30,31]. The friction grippers consist of two or more opening and closing fingers (micro-tweezers) to manipulate SMT parts. Several types of actuation can be adopted for these grippers, which include pneumatic, piezoelectric, Shape Memory Alloys (SMAs), or electric actuators [32,33,34,35,36]. In addition to these types of micro-grippers, there are also hybrid micro-grippers that combine different grasping mechanisms or actuations to achieve better performance [37,38].

In this context, this paper presents the design and development of a multi-mode hybrid micro-gripper devoted to manipulate and assemble a wide range of micro- and meso-SMT components with different dimensions and proprieties. It exploits two different handling technologies: the vacuum and friction. Section 2 describes the mechanical design of the developed hybrid micro-gripper. Section 3 presents the gripper control framework and the manipulation performance.

## 2. Design of a Multi-Mode Micro-Gripper

The design of the micro-gripper for the SMT component manipulation embodies a multi-mode approach: it is realized in the form of a hybrid micro-gripper, which combines vacuum technology with mechanical side-gripping technology. In particular, the designed gripper embeds two gripping technologies:

- The vacuum technique, in the form of miniaturized suction cups (gripper nozzle);
- The mechanical gripping, employing two properly designed parallel fingers.

**Figure 1:**
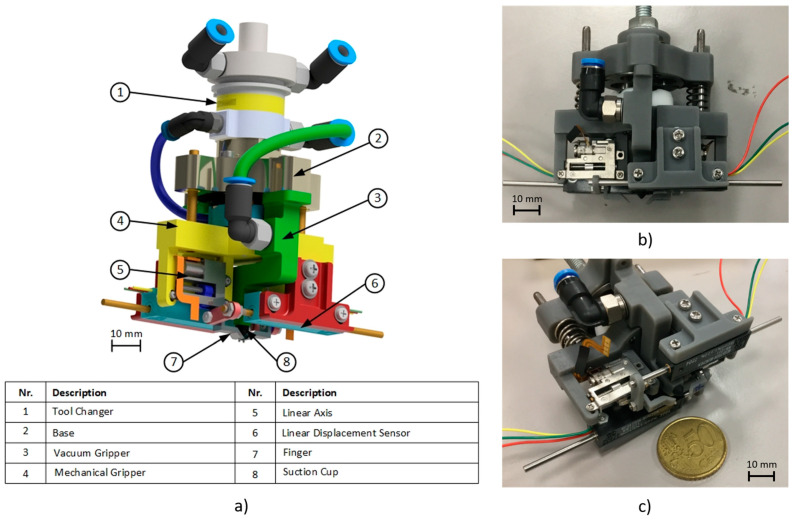
CAD model of the designed micro-gripper (**a**), front view of the realized prototype (**b**), and axonometric view (**c**).

The CAD design and the 3D-printed prototype of the gripper are shown in Figure 1.

The hybrid micro-gripper was developed for the micro-manipulation and micro-assembly of SMT components on Printed Circuit Boards (PCBs). It can be mounted as an end-effector of a robotic system devoted to PCB assembly. The advantage of the designed gripper is its flexibility in manipulating a wide range of different types of SMT components in shape and size. Indeed, the gripper can manipulate (i.e., pick, hold, and release), with high accuracy, SMT components in the planar size range of 0.5 × 0.5 mm–8 × 8 mm with a variable height between 0.30 mm and 5 mm. Thanks to the double technologies, it can firmly hold the component with a bilateral constraint along two orthogonal axes (i.e., *X*- and *Z*-axis). The gripper was designed to ensure a maximum grasping and holding bilateral force of 0.2 N along the two orthogonal axes. This specification also allows the gripper to be used for soldering operations by external automatic/manual soldering systems. Indeed, it was estimated that the forces exerted by the soldering tools on the component during a manual/automatic soldering operation is in the range of 0.05 ÷ 0.2 N, based on the technology adopted. Using this gripper for the SMT component assembly, the soldering can be executed manually by the human operator with a soldering iron while the component is held in place by the robot using the gripper (i.e., collaborative task) or automatically by an external automatic soldering iron system, reflow, or curing tools. To perform this operation, the strategy is to grasp the component both with the micro-suction cup and with the mechanical fingers, according to the shape and size of the manipulated component. This strategy ensures a more precise and firmer grasping. Another important issue considered in the design of the micro-gripper was the working temperature. Since during the soldering operation, the temperature around the fingers can reach up to 200 °C, the finger material was designed to comply with this requirement.

### 2.1. Components and Subsystems

**Figure 2:**
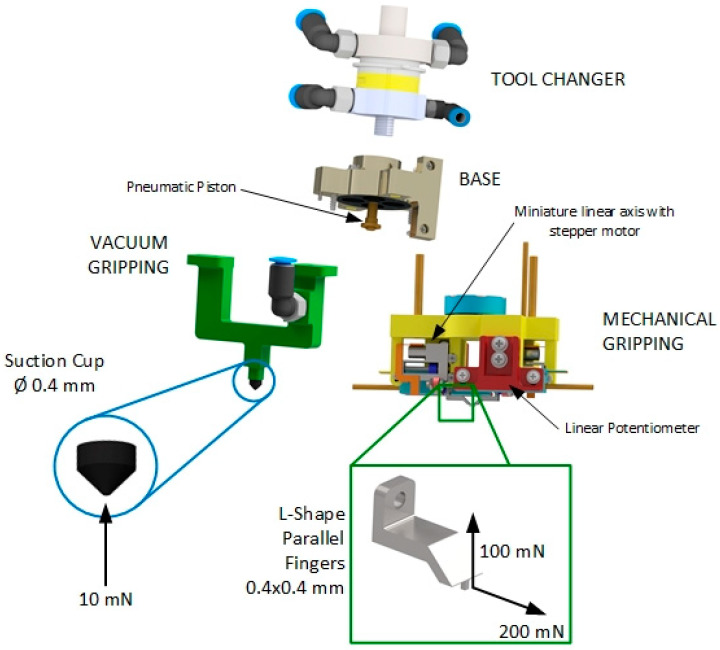
The Innovative Micro-Gripper and its functional sub-assemblies.

The designed hybrid micro-gripper consists of four sub-assemblies, as shown in Figure 2: the tool changer, the pneumatic cylinder, the vacuum gripper, and the mechanical gripper.

The components that characterize the performances of the whole system are the miniature suction cup, the pneumatic piston, the miniature stepper motor, and the linear potentiometers.

The vacuum gripping sub-assembly is equipped with an interchangeable miniaturized rubber suction cup and a suitable holder to mount it, both manufactured by Micro-Mechanics and shown in Figure 2. Several rubber cups with different internal and external diameters in the range of 0.1 ÷ 3 mm can be mounted to manipulate components of different sizes. The gripper prototype was equipped with a rubber suction cup with an inner diameter of 0.4 mm and an external diameter of 0.6 mm. To generate the vacuum in the suction cup, a compact Venturi effect system by PIAB (i.e., piCompact Micro Si with a maximum vacuum pressure of −0.8 bar) was used, including a 3-2 solenoid valve for activating the vacuum, a vacuum switch, and an analog pressure/depression sensor.

**Figure 3:**
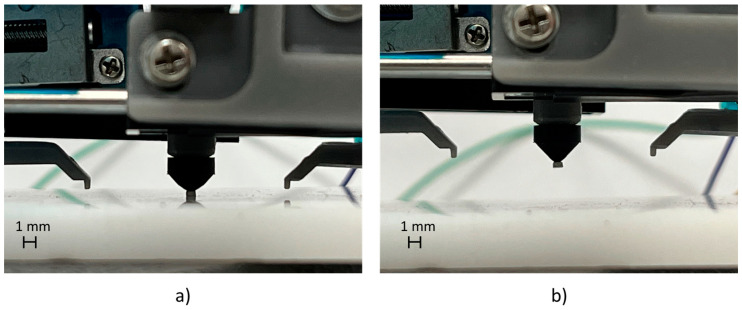
Pick-and-release test cycle of a 1005 resistor: (**a**) picking position; (**b**) upward movement.

**Figure 4:**
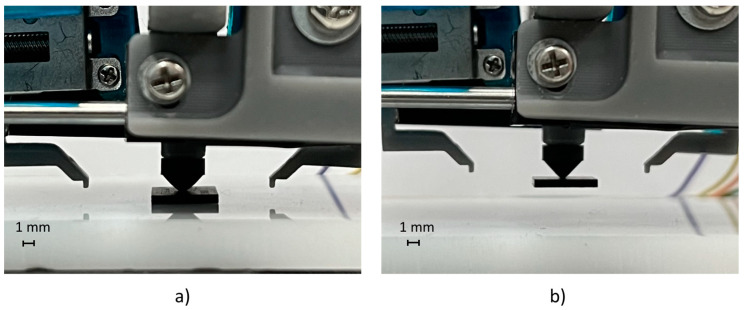
Pick-and-release test cycle of a BGA package: (**a**) picking position; (**b**) upward movement.

Preliminary experiments were carried out to validate the manipulation of SMT components exploiting vacuum technology. The following SMT components were tested: 1005 resistor (1 × 0.5 mm), 2012 resistor (2 × 1.25 mm), and BGA package (8 × 6 mm). A total of 30 tests of pick-and-release operations for each component were executed, generating the vacuum on the suction cup of the gripper through a 3-2 electro-valve. These preliminary trials showed a reliability of 100% in picking and releasing components in the dimensional range of 0.5–8 mm. Figure 3 and Figure 4 show the manipulation sequence on the 1005 resistor and the BGA package.

Another critical component is the pneumatically actuated monostable cylinder, representing the vertical axis of the gripper fingers. Indeed, the cylinder piston can move up the fingers, providing compressed air into its chamber, and it can move down them, restoring the atmospheric pressure into the chamber and under the elastic force action of the springs.

The designed cylinder is not equipped with piston seals to reduce friction and the stick-slip effect as much as possible. Indeed, when compressed air is provided into the chamber, an air cushion between the piston and the cylinder barrel is present, reducing the friction. The drawback is a little air leak that increases the air consumption.

A proportional pressure regulator and a 3-2 solenoid valve control the motion of the cylinder piston. This solution allows an open-loop motion control and a closed-loop lifting force control of the vertical axis.

**Figure 5:**
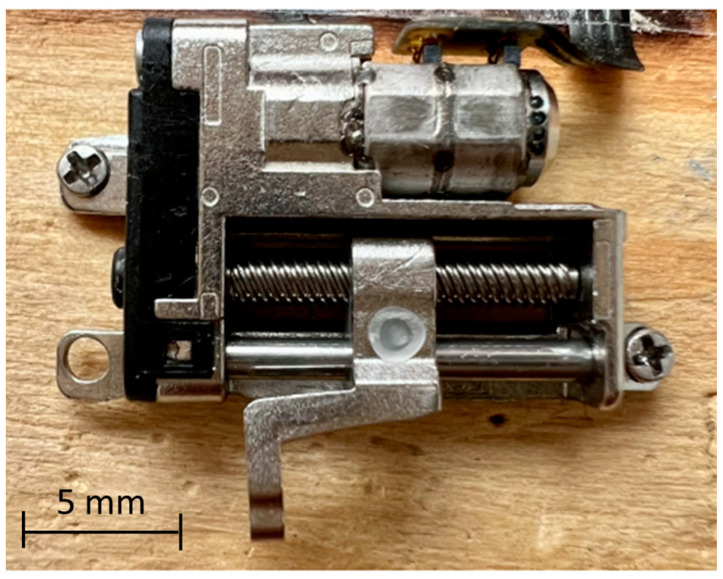
Miniature stepper motor.

The micro-gripper incorporates two low-cost miniature linear axes with stepper motors [39], as shown in Figure 5. These linear axes are equipped with a transmission system for reducing and transforming the motion from rotary to linear by gears coupled with screws and nuts. The axis specification is reported in Table 1.

The gripper incorporates two position sensors in order to have feedback on the position of the two fingers and to implement closed-loop motion control. The chosen sensors are the MM10 linear potentiometers by Megatron, with a total resistance of 5 kΩ, resistance tolerance of ±10%, and independent linearity under ±1%. The theoretical resolution is almost infinite, but the actual resolution of the measurement system can be obtained from the resolution of the ADC and the full stroke of the potentiometer. The potentiometer is powered with 3.3 V, and its full stroke is 11 mm. The ADC has 12-bit resolution, resulting in a measurement resolution of the sensor equal to 2.68 µm for both fingers.

The fingers were designed in order to manipulate components in the range of 0.5 × 0.5–8 × 8 mm. They are interchangeable, and two different designs were proposed: the former presents an L shape, and the latter presents a T shape, with both having a grasping surface of 0.4 × 0.4 mm (as shown in Figure 2).

### 2.2. Multi-Modal Operational Mode

This section describes the three possible operational modes for the designed micro-gripper. These are characterized by using only one technology for picking the SMT component or both. In particular, the designed micro-gripper allows

1. Mechanical picking;
2. Vacuum picking;
3. Hybrid picking (both vacuum and mechanical picking).

**Figure 6:**
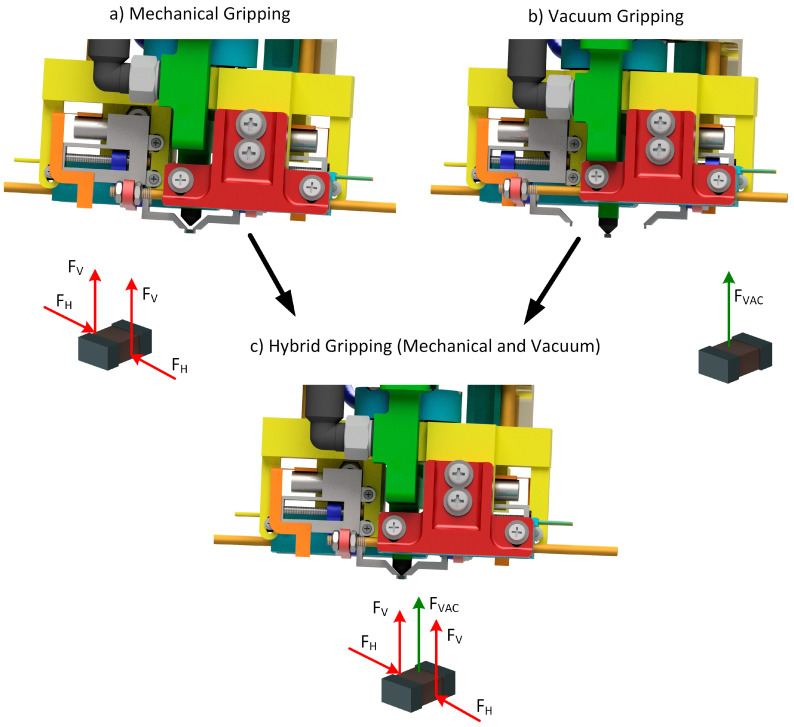
The three operational modes for the hybrid micro-gripper with the corresponding free-body diagrams of the forces acting on the gripped component: F_H_ represents the horizontal force exerted by the finger; F_V_ represents the vertical force related to F_H_ due to friction; and F_VAC_ represents vertical force due to the suction cup.

Figure 6 illustrates these three operational modes with the corresponding free-body diagrams of the forces acting on the gripped component.

The first operational mode exploits the two parallel fingers to pick the SMT component. Since the gripper is mounted on the end-effector of a robotic system, the operational steps for the mechanical picking task are

- Position 1 (Home): the robot moves the gripper to the X and Y coordinates of the component barycenter while the robot’s Z-axis is lifted. The fingers are opened, and the gripper pneumatic axis is in the bottom position.
- Position 2 (Approach along the Z-axis): Starting from Position 1, the robot’s *Z*-axis is lowered until the fingers touch the work surface. This phase is useful to reference the plane on which the component must be picked.
- Position 3 (Up): The robot’s *Z*-axis is raised from 50 to 100 µm (with the fingers opened) according to the component height. This strategy ensures that the component is picked 50/100 µm above its bottom surface, avoiding interference between the bottom surface of the gripper fingers and the releasing substrate (i.e., the PCB) during the releasing of the component.
- Position 4 (Mechanical picking): The two fingers of the gripper are closed, and the component is picked. The fingers are moving, exploiting the designed closed-loop motion control for the finger axes. This position is shown in Figure 6a.

For the vacuum picking, Position 1 is repeated in the same way, while Position 2 is repeated until the tip of the micro-suction cup gets in contact with the SMT component. In this case, Position 3 is unnecessary, and Position 4 (Vacuum picking) is obtained, activating the vacuum inside the suction cup by the 3-2 solenoid valve. This position is shown in Figure 6b, where it is possible to see the component picked by the micro-suction cup while the fingers are opened.

The hybrid operational mode is implemented, combining the two modalities described above. In particular, after Position 4 (Mechanical picking), Position 5 (Hybrid picking) consists of lifting the gripper pneumatic axis until the component is placed on the micro-suction cup. The 3-2 solenoid valve activates the vacuum, and the micro-suction cup grasps the component. Exploiting this hybrid operational mode, the component is grasped precisely and firmly. This position is shown in Figure 6c.

The above-described procedures are for component picking in the different operational modes; dual procedures can be exploited in reverse order to release the components on the PCB substrate after the soldering operation.

## 3. Gripper Controller

### 3.1. Components

**Figure 7:**
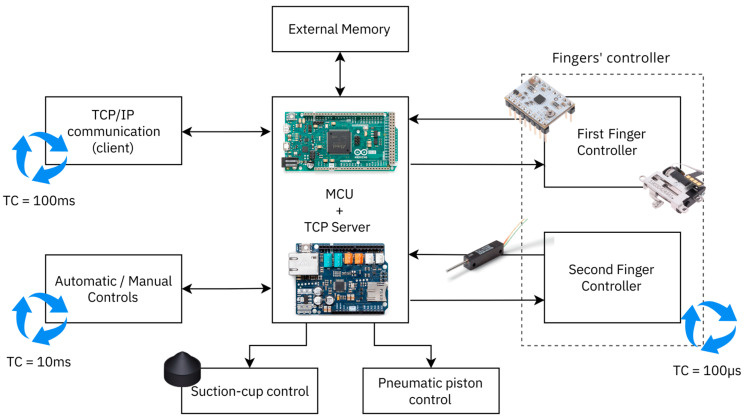
Functional block scheme of the micro-gripper controller.

The controller was designed to control and manage all the micro-gripper functionalities, ensuring compliance with the project specifications reported in the previous section: open-loop control of the vertical lifting piston of the gripper fingers, on/off control of the suction vacuum, closed-loop position control of the two fingers independently, Ethernet TCP/IP communication for receiving commands and sending status messages, and external manual driving via the control panel or automatic one via digital signals. For this reason, the controller comprises several subsystems and components, as can be seen in the functional block scheme in Figure 7.

The core of the control is the Microcontroller Unit (MCU), which consists of an Arduino Due, equipped with an Ethernet Shield v.2. The choice of this MCU, in addition to its great flexibility, modularity, and ease of use, depended mainly on the high computational speed, as it runs at 84 Mhz, the high number of digital I/O pins, and the availability of a 12-bit ADC. Its high clock frequency made it possible to create a pseudo-real-time multi-thread control system with different cycle times and to use its timers to command drivers both in open and closed loops. Three threads were created: the first one for the TCP/IP communication running with a period of 100 ms, the second one for the fingers’ controls, running with a period of 100 µs, and the last one for the external command signals that read input digital signals or the pushbutton states, running with a period of 10 ms.

An external EEPROM was used to store the basic configuration parameters to handle possible undesirable effects caused by a power failure or controller shutdown and restart. The 256 K I2C CMOS Serial EEPROM 24LC256 from Microchip was chosen.

The functionality of the gripper can be managed in three different modes: manual mode via manual pushbuttons, automatic mode via digital input signals, and communication mode via TCP/IP communication. The communication mode is always enabled, while a selector switch was equipped on the controller to switch between automatic and manual modes. The signal inputs for automatic handling mode were equipped with opto-isolated static relays to decouple the gripper control system circuit to the external driving circuit (e.g., robot controller, PLC, …) where the digital control signals were output.

The suction vacuum and the vertical piston position were controlled by two normally closed 3-2 solenoid valves, driven, respectively, by two static opto-isolated relays that bring the correct voltage to the two solenoids. Two digital outputs of the microcontroller were used to change the state of the static relays as on/off controls.

Finally, the elements that make up the finger controllers were two STSPIN220 Low-Voltage Stepper Motor Drivers, two precision linear potentiometers, and two linear axes mechanically connected to the gripper fingers, respectively, composed by a micro-claw pole stepper motor and a mechanism of transmission and transformation of motion (i.e., gearbox and screw drive). The chosen drivers allowed the stepper motors to be driven with the STEP/DIR logic at a high frequency and to increase the step resolution using a micro-stepping down to 1/256th of a step. In this paper, a micro-stepping of 1/4th was used. The resulting linear step of the linear axes was 6.25 µm/step. The precision linear potentiometer provided position feedback for the closed-loop control through PID controllers.

### 3.2. Control Algorithm

The gripper components that we wanted to be controlled were the piston for movement along the vertical axis, the suction cup, and the fingers. As said, an open loop and an on/off control were implemented, respectively, for the first two components by exploiting two solenoid valves and two relays for power conversion. Differently, a more advanced control was implemented for the fingers that allowed both open-loop and closed-loop driving, exploiting the previously described driver with a STEP/DIR driving mode. An open-loop control algorithm with a trapezoidal speed profile following was implemented, exploiting the method described in [40]. The method allowed the generation of a real-time trapezoidal/triangular velocity profile for stepper motors with an MCU. Then, knowing the transmission ratio of the linear axis, it was possible to calculate the rotative motion law of the motor to perform the desired linear movement of the finger. The trapezoidal velocity profile was completely defined by four of the following parameters: total displacement ∆S [rad], total motion time ∆T [s], acceleration A [rad/s^2^], deceleration D [rad/s^2^], maximum speed V_max_ [rad/s], and $\lambda_{i}$, with i=1...3 where $\lambda_{i}$ is the ratio between the i-th section time of the law of motion and ∆T, and $\lambda_{1}+\lambda_{2}+\lambda_{3}=1$. The profile was approximated with a sequence of speed steps. Each step has a certain duration, corresponding to the interval between one step of the motor and the next. As the speed increased (or decreased), i.e., in the ramps of the speed profile, the delay between consecutive steps decreased (or increased). Instead, the delay remained constant in the profile section at a constant speed. The algorithm can be implemented by exploiting the timers of the MCU. Through appropriate approximations, the following equations were obtained:

(1) $$\delta t=\frac{c}{f}\left[s\right],$$

(2) $$\omega =\frac{\alpha \cdot f}{c}\;\left[\frac{rad}{s}\right],$$

(3) $$c_{0}=f\cdot \sqrt{\frac{2\cdot \alpha }{\dot{\omega }}}\;,$$

(4) $$c_{i}=c_{i-1}-\frac{2\cdot c_{i-1}}{4\cdot n_{i}+1}\;,$$

(5) $$n=\;\left[\frac{\omega^{2}}{2\cdot \alpha \cdot \dot{\omega }}\right],$$

(6) $$\left(n_{1}+0.5\right)\cdot {\dot{\omega }}_{1}=\left(n_{2}+0.5\right)\cdot {\dot{\omega }}_{2}.$$

Equation (1) describes the delay between consecutive steps δt, obtained with a 32-bit timer count *c* [-], with a timer frequency *f* [Hz]. Equation (2) expresses the motor speed obtained with a timer count *c* and a motor step angle α [rad/step]. Equations (3) and (4) express the first count of the timer and the i-th subsequent counts needed to obtain the speed ramp, where i is the sequence index, $\dot{\omega }$ [rad/s^2^] is the acceleration in the first section, and $n_{i}$ determines the acceleration/deceleration. It increments with i for constant acceleration, i.e., $n_{i}=i\;,\;i=1,\;2,\;\ldots$, while it starts from a negative value and increments for constant deceleration. Indeed, Equation (4) with $n_{i}=i-m\;,\;i<m$ can be used to ramp any speed down to stop in the final steps of a movement of m steps. Equation (5) expresses the step number n as a function of speed and acceleration and can be used to obtain the number of steps required to reach a desired speed with a desired acceleration, starting from a standstill motor. The value thus calculated also corresponds to the number of steps required to reach zero speed, starting from velocity ω with desired deceleration $\dot{\omega }$ (in absolute value). The number of steps needed to reach a given speed is inversely proportional to the acceleration. This makes it possible to change the acceleration at a point on the ramp by changing the step number n in the ramp algorithm, by using Equation (6), where a half-step is added to n-values for better accuracy.

Then, starting from the parameters that characterize the law of motion, the total number of steps to be performed in time ∆T is first derived to make the total displacement ∆S: n=∆S/α. Then, Equation (5) derives the number of acceleration and deceleration steps from the values of maximum speed, acceleration, and deceleration, which are imposed as parameters of the law of motion. Finally, the number of steps of the constant velocity section is calculated using subtraction. During the movement, only the time between consecutive steps is calculated, and it is defined by the numerical sequence of the timer count c.

The open-loop control algorithm takes advantage of this method of generating speed ramps to produce a trapezoidal law of motion in real-time for the fingers, in which a 32-bit timer unit manages the generation of the train of pulses (i.e., a square wave with a duty cycle of 50%) input to the motor driver, with a variable frequency, calculated through the algorithm at each timer interrupt. The maximum driving frequency of the open-loop control was 42 [MHz], but it was limited by the maximum frequency (i.e., maximum speed of the stepper motor) allowed by the motor, equal to 24 [kHz]. During the constant speed section, the algorithm was suspended for the desired number of steps, calculated in advance, knowing the number of steps needed to perform the motion and the number of steps needed for the acceleration and deceleration phases.

Instead, closed-loop position control was performed using a PID controller, with trapezoidal law of motion tracking. Position feedback was obtained using the precision linear potentiometer.

#### 3.2.1. Feedback Signal Filtering and Sensor Calibration

Before the closed-loop control could be developed, it was necessary to analyze the position feedback acquisition system. As mentioned, two potentiometers with a measuring range of 11 mm acquired the position feedback for the two fingers. As introduced in Section 2.1, the 12-bit ADC included in the Arduino Due board was used for digital conversion, and then the systems acquisition resolution obtained was 2.68 µm. The first step that needed to be carried out was the analysis of the feedback signal to evaluate the measurement noise. Raw data were acquired from different positions of the two linear potentiometers placed on the fingers to evaluate the standard deviation of the measurement. The chosen positions were the closed pose, the open pose, and a position in the middle of the full range of movement. Table 2 shows the values of the standard deviation measured for the two potentiometers in the different positions. It is worth noting that the maximum value was obtained in the open position when the voltage signal was lower. A second-order low-pass filter was designed to reduce the maximum standard deviation. A 3 dB cutoff frequency of 50 [Hz] was selected. The standard deviation values obtained from the same filtered signals as before are shown in Table 3. By comparing the two tables, it can be seen that the standard deviation was reduced by 87% for the first finger and by 89% for the second one. Knowing the resolution of the acquisition system, the maximum standard deviations of the filtered potentiometer signals can be converted into micrometers, obtaining a value of 6.57 [µm] for the first finger and a value of 6.52 [µm] for the second one.

To perform the sensor calibration with a high resolution, it was necessary to verify the maximum stroke of the linear axes. An absolute linear indicator (i.e., Mitutoyo Digimatic Indicator ID-C112B No. 543-250B) with a linear resolution of 0.001 [mm], a measuring range of 12.7 [mm], and an accuracy of 0.003 [mm] was used as an external measurement system. The stroke was obtained by measuring the linear displacement of the axis from the first limit switch (corresponding to the fully opened position) to the second one (corresponding to the fully closed position) for both fingers, resulting in a maximum range of movement of 9119 [µm] for the first finger and 9086 [µm] for the second one.

Finally, the linear calibration of the sensor was performed by developing an automatic routine into the microcontroller. The controller acquired raw values of the potentiometers from the ADC at the extremes of the motion range (i.e., limit switches) and then computed the linear calibration, knowing the range of movement of the fingers saved into the memory.

#### 3.2.2. Mechanical Backlash

**Figure 8:**
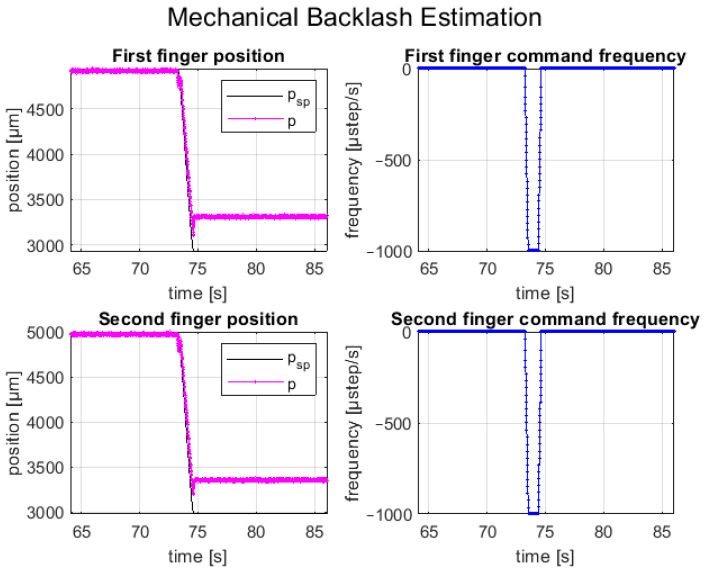
Example of mechanical backlash during an open-loop movement.

An experimental test was carried out to evaluate the mechanical backlash of the two linear axes of the fingers. The test for both axes involved several repetitions of a first initialization open-loop movement in one direction and a second open-loop movement in the opposite direction. This choice was made because the preliminary experiment showed that the mechanical backlash was more significant after changing the movement direction; therefore, it represented the worst condition. To avoid step loss due to the load torque, the tests were carried out by setting the maximum step frequency equal to 1 kHz, to assure a suitable motor torque. In this way, the steady-state error position of the tests was mainly caused by the mechanical backlash of the linear axis. Furthermore, a short displacement was commanded, in such a way that the error related to the theoretical step angle tolerance was reduced, and the main component of the steady-state error was the mechanical backlash. Figure 8 shows the setpoint position (*p*_sp_), the feedback position (*p*), and the commanded step frequency of both fingers during a trial, consisting of an opening movement with a relative displacement of 2 mm and $\lambda_{1,2}=0.25$. It is worth noting that the feedback positions do not reach the theoretical positions due to the mechanical backlash of the linear axes.

Different trials were carried out, and the maximum mechanical backlash measured for both finger axes is reported in Table 4.

#### 3.2.3. Closed-Loop Control Algorithm Development

Stepper motors are prone to losing steps, and the backlash axis causes position errors. These issues can be prevented by oversizing the motor or implementing closed-loop control with position feedback. Closed-loop control offers the advantage of detecting and correcting position errors while optimizing torque selection and energy consumption.

One closed-loop control technique is step-loss compensation, where the motor operates in an open loop while position feedback tracks the movement. Missing steps are counted and executed at the end of the motion profile to reach the desired position. However, this method only corrects the position at the end of the profile.

Load position control continuously monitors the position and generates an error signal for real-time adjustments during the motion profile. While the motor operates in micro-stepping mode as if it were in an open loop, it closely follows the motion profile.

Field-oriented control, the most advanced method, treats the motor as a two-phase brushless motor. Instead of supplying the maximum current, a control loop with a PID controller determines the required torque for precise movement, deriving the current magnitude from it. This method improves efficiency, reduces heating, extends motor life, and eliminates resonance issues. It is also known as vector control, which involves transforming the coordinates of the motor’s electric and magnetic fields to a rotating reference system aligned with the rotor. This allows AC motors to be driven similar to DC motors with brushes. This method is also called vector control in the literature. A comprehensive explanation of field-oriented control can be derived from [41,42].

Sensor-less control relies on back-electromotive force (back-emf) to determine the rotor position. Some cases of study were analyzed [43,44,45]. Back-emf is used to estimate and control the load angle, aiming to maximize torque output. However, this control method is unsafe since back-emf can only be measured at zero-crossing points of the current, and accurate estimation relies on a reliable motor model. Also, it is challenging to implement it and lacks certainty in rotor position determination.

Considering the complexity and model requirements of existing closed-loop control methods, a different technique was introduced in the paper. The developed closed-loop control strategy does not require detailed knowledge of the motor’s physical model and its design parameters, making it suitable for use with low-cost stepper motors where some specific design data may be not provided by the manufacturer while allowing high positioning precision suitable for the micro-manipulation of SMT components. It employs position feedback control with a PID controller, taking the position error as input and generating the step pulse command for the stepper motor, aided by speed feedforward. The speed is converted to a stepping frequency and sent to a low-cost stepper motor driver using the STEP/DIR interface. The driver limits the maximum current and maintains the desired current level, defining the micro-stepping position through an internal closed-loop circuit. This technique offers an alternative approach to achieve effective closed-loop control without relying heavily on complex motor models.

**Figure 9:**
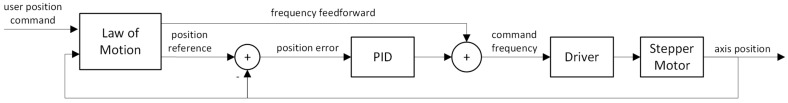
Block scheme of the axis position controller.

The closed-loop position control scheme was designed and implemented to synchronize and control the motions of the fingers with high precision, avoiding position errors due to mechanical backslash of the axes and step loss of the stepper motors. As said, a decentralized control was realized for the two axes of the fingers, implementing a position control closed-loop for each axis, as shown in Figure 9. The motion law generation block, the feedback acquisition, and the generation of the control variable were carried out internally in the Arduino Due microcontroller. The block that defines the trapezoidal law of motion consists of a state machine, which outputs the desired value for position and velocity as time changes. The states represent the different sections of the law of motion (i.e., acceleration, constant speed, deceleration, and stop), and the transition between one and the following depends on the elapsed time. The position error between the theoretical position of the finger axis and the actual one measured by the linear potentiometer is input to the PID controller. Moreover, an anti-windup compensation for the integrator term of the PID controller was implemented with back-calculation. The controller only acts if the error exceeds a previously set control positioning tolerance, which depends mainly on the position feedback measurement maximum error (i.e., maximum standard deviation of the position measurements), the step size of the motor, and the mechanical backlash. The control tolerance prevents unwanted chattering in the control variable (i.e., the driver input frequency), which could damage the motor and reduce its lifetime. The control tolerance was set to ±50 µm after carrying out experimental closed-loop motion tests.

A speed feedforward action was also introduced to improve the setpoint tracking; therefore, the step frequency of the motor was given by the sum between the output of the PID controller and the feedforward action. In closed-loop circuits, the maximum driving frequency is lower than in open-loop circuits and is equal to 15 [kHz], obtained by experimental tests, above which the synchronization of the two fingers cannot be guaranteed, as a delay of the controller can be observed. The MCU generates the driving square wave at the desired frequency by exploiting a timer unit with a maximum output frequency of 42 MHz and is sent as input to the STEP pin of the driver. The movement direction is commanded with the DIR pin of the driver, based on the sign of the command frequency. Finally, position feedback is acquired by the ADC, which measures the digital voltage value of the linear potentiometer.

A suitable strategy was adopted to cope with external disturbances acting on the axes or the fingers, such as an external force moving a finger when it is stopped or a finger colliding with the external environment and stopping when it is moving. If the movement was already initiated, the new position and speed, outputs of the law of motion block, are computed; then, a comparison between the starting absolute position error and the actual error is made. If the error increased, a new law of motion is computed. In case the movement was not started yet (i.e., the position reference is constant, and the feedforward action is null), for little position errors, the PID controller is responsible to reject the disturbances. If the position error leads the PID controller output over the maximum starting frequency of the stepper motor, a new law of motion is computed starting at the actual axis position to avoid the stepper motor stall.

When the position error increases beyond a stall threshold, set equal to the estimated mechanical backlash of the axis, the motion is stopped to prevent damage. This method, known as collision or stall detection, is very useful to prevent damage.

**Figure 10:**
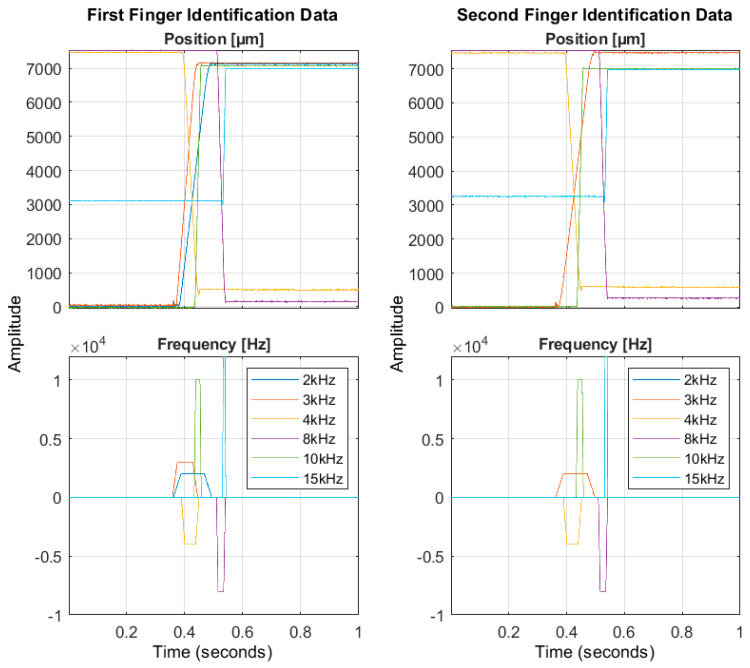
Identification trials for both fingers.

**Figure 11:**
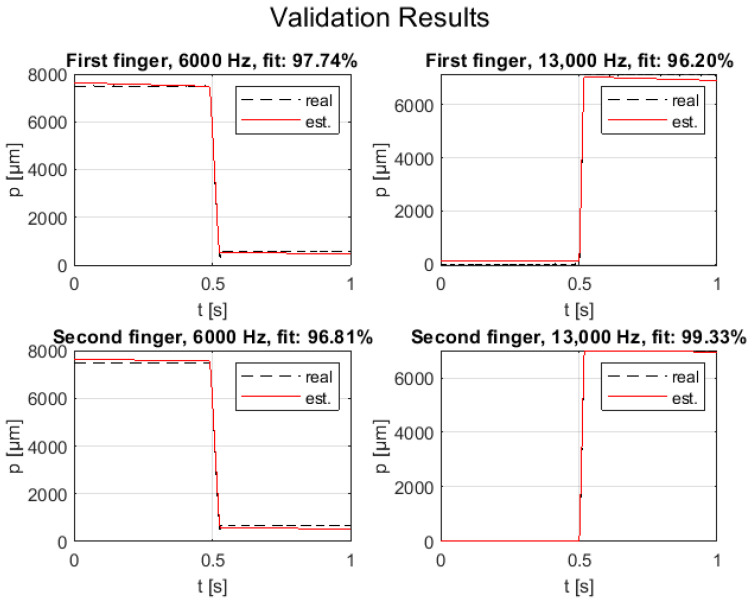
Validation trials for both fingers.

To fine-tune the PID controllers of the axes, it was necessary first to perform the linear system identification test. A time-domain impulse-response identification was performed for each axis, carrying out several open-loop motion cycles. In particular, for each motion cycle, a trapezoidal speed was set as system input, and the reached output positions were acquired with a sample time of 0.5 ms using the calibrated potentiometer. A total of eight cycles were executed for each finger axis, varying the maximum motion speed in the range of 3–24 mm/s and the driving time in the range of 0.4–3 s. Merging six test trials, chosen randomly among those performed, first-order transfer functions were estimated for the axes using the MATLAB System Identification toolbox. Then, the validation of the system identification was carried out by exploiting the remaining two trials. Figure 10 shows the chosen identification trials for both axes: the frequency data represent the system input, and the position data represent the system output. Figure 11 shows the position estimation by the identified model (i.e., first-order transfer function response, expressed in Equation (7)) using the input data of validation trials for both axes. It is worth noting that the model response fits the system dynamic of 96% in the worst case for both axes. The first-order model constants for the finger axes are reported in Table 5 and the transfer function is:

(7) $$P\left(s\right)=K\cdot \frac{1}{1+T\cdot s}$$

Finally, the tuning of the PID controllers was performed using the MATLAB PID tuner toolbox. A PI controller was selected for both axes, setting the robustness to the maximum value to avoid unwanted overshoots and increasing the performance as much as possible to improve the setpoint tracking and noise rejection trade-off. The PID controller values for the two finger axes are reported in Table 6 and the transfer function is:

(8) $$R_{PID}\left(s\right)=K_{p}+\frac{K_{i}}{s}\cdot K_{d}\cdot s$$

#### 3.2.4. Control Performance Assessment

Experimental tests were executed to assess the positioning and synchronization performance of the closed-loop motion control designed for the gripper fingers.

Firstly, the control capability of setpoint tracking was assessed with experimental tests. Several repetitions of picking and releasing cycles of sample SMT components (i.e., a BGA package with the size of 8 × 6 × 0.8 mm and a 1005 resistor with the size of 1 × 0.5 mm, both with a prismatic shape) were executed. The selected samples represent the minimum and maximum dimensions of the components that the designed gripper can handle. For each test, the setpoint tracking error and the time delay error (i.e., finger motion synchronization error) were computed, and finally, the error means and the standard deviations were calculated.

**Figure 12:**
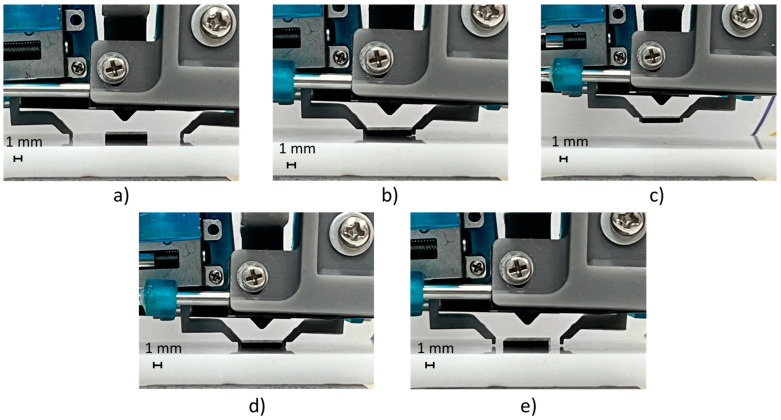
The picking and releasing test cycles of the BGA package: (**a**) initial position; (**b**) picking position of the fingers; (**c**) upward movement; (**d**) downward movement; and (**e**) releasing position of the fingers.

**Figure 13:**
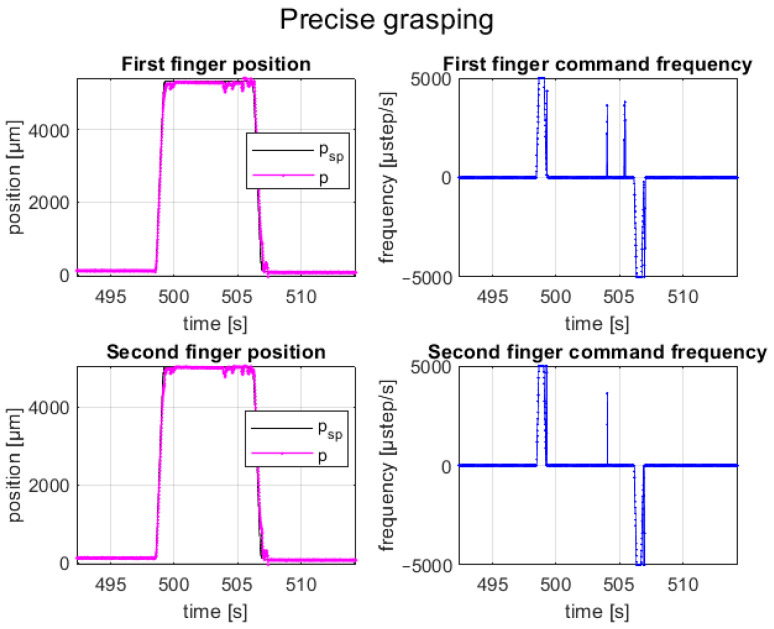
Setpoint position (black line), feedback position (red line), and frequency command (blue line) for each finger during the grasping and releasing tests of the BGA package.

A test cycle emulated the pick-and-place task of an SMT component; it composed of a closed-loop motion of finger axes in precise positions (e.g., obtained by identifying the component size with a vision system or knowing the component size by data sheet) for picking the component, an upward movement of the gripper fingers (e.g., by a robot or gripper piston), a downward movement (e.g., by a robot or gripper piston), and a closed-loop motion of finger axes in precise positions for releasing the component on the substrate. The grasping and releasing cycles of the BGA component are shown in Figure 12, and its setpoint tracking plots are shown in Figure 13. The represented trial consisted of a relative displacement (opening and closing) of 5.2 mm for the first axis and 4.9 mm for the second axis, with a maximum step frequency of 5 kHz for both fingers in both directions, with $\lambda_{1,2}=0.25$.

**Figure 14:**
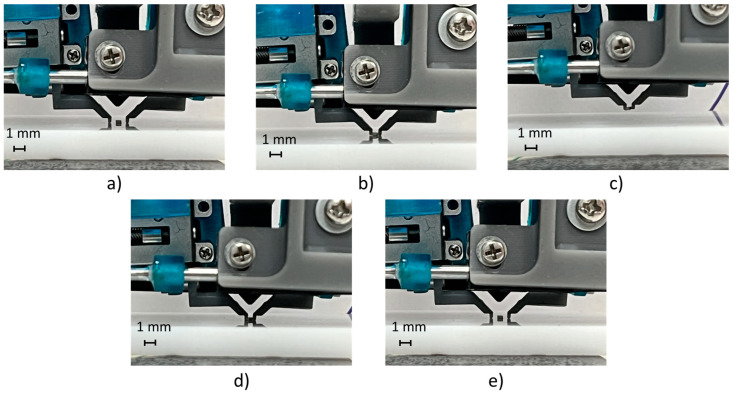
The picking and releasing test cycles of a 1005 resistor: (**a**) initial position; (**b**) picking position of the fingers; (**c**) upward movement; (**d**) downward movement; and (**e**) releasing position of the fingers.

**Figure 15:**
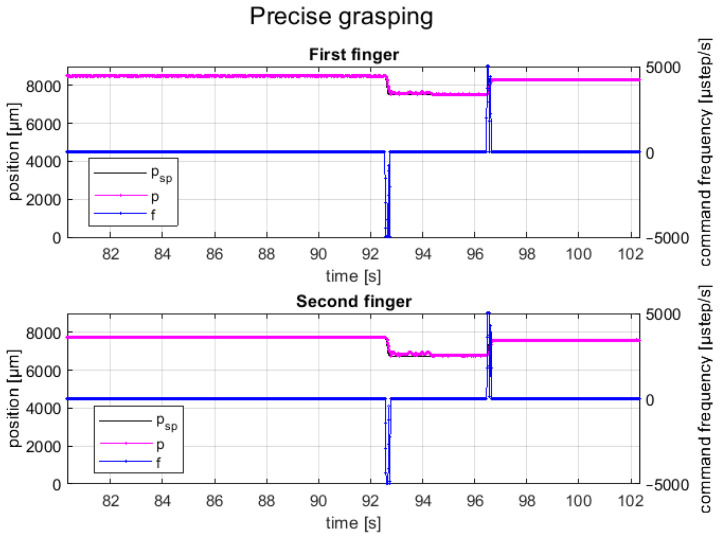
Setpoint position (black line), feedback position (red line), and frequency command (blue line) for each finger during the grasping and releasing tests of the 1005 resistor.

Instead, Figure 14 shows the picking and releasing cycles of a 1005 resistor component, which is the smallest component that the gripper can manipulate, while the related setpoint tracking plots for both fingers are shown in Figure 15. In this case, the trial consisted of a relative displacement (opening and closing) of 0.8 mm for each finger, with a maximum step frequency of 5 kHz for both fingers in both directions, with $\lambda_{1,2}=0.25$.

It can be seen that the feedback position of the closed-control faithfully follows the setpoint position for both finger axes with a positioning error of less than ±50 µm (i.e., control tolerance); moreover, the motions of finger axes are completely synchronized with a time delay error less than 30 ms.

The second test consists of several repetitions of picking and releasing operations, using the stall detection option to detect the component in the picking phase and collision with the environment in the releasing phase. This option can be enabled independently for the two fingers or dependently, i.e., in the presence of a stall detection for a finger, both are stopped. The first option was selected to perform this test.

**Figure 16:**
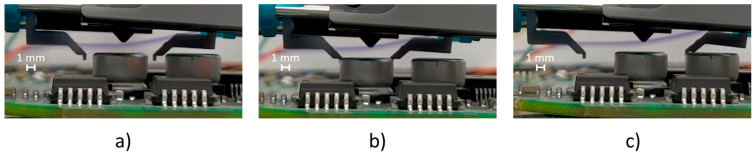
The picking and releasing test cycles with the stall detection option enabled: (**a**) initial position; (**b**) grasping position of the fingers; and (**c**) collision of a finger with the capacitor during the releasing phase.

**Figure 17:**
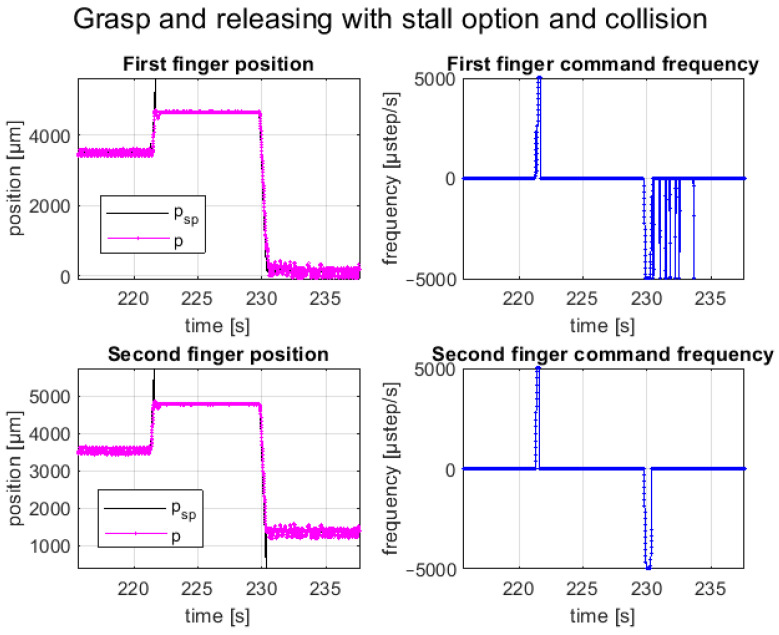
Position and frequency command of picking and releasing test cycles with the stall detection option enabled independently for the two fingers.

A total closure of the fingers was commanded to pick the component (i.e., electrolytic SMT capacitor with a size of 6 × 8 mm and a cylindrical shape); once the fingers collided with the component, they stopped and held the component in place due to the holding torque of the motor. The releasing phase was evaluated by simulating a collision of a finger with a component mounted on an electronic board during the full opening of the fingers. Once the finger collided with the obstacle, the controller recognized the stall and stopped both fingers in place. The picking and releasing cycles of the sample component with the stall detection option enabled are shown in Figure 16. The position tracking plots are shown in Figure 17. The represented trial consisted of a movement with a maximum step frequency of 5 kHz for both fingers in both directions, with $\lambda_{1,2}=0.25$. As explained before, a full close motion was commanded, resulting in a relative movement of 5.6 mm, as the starting position was not fully opened. Instead, for the releasing phase, a movement of the full stroke of 9.1 mm was commanded. It is worth noting, that when the stall occurred, the setpoint position (black line) increased until the stall threshold was reached, and then, the setpoint was fixed to the current position and the law of motion was stopped.

**Figure 18:**
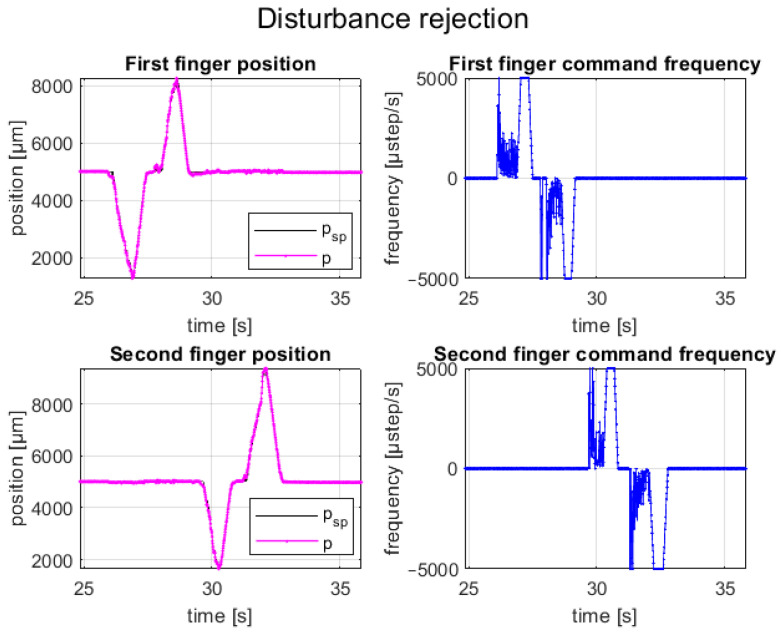
Finger position and control variable of a disturbance rejection test cycle.

Another test performed concerns the disturbance rejection of the closed-loop control. External forces were applied in two different time instants to the fingers, and the system dynamics were observed. The controller reacted by recalculating the law of motion until the external disturbance disappeared, returning the system to the setpoint positions. Figure 18 shows the position tracking during the disturbance rejection test. The behavior was the same for both fingers and for both open and close directions. First, when the external force was applied, the controller computed a new law of motion until the error increased; then, as soon as the external force was removed, the controller restored the setpoint position following the last computed law of motion, as shown in Figure 18. The new law of motion was computed based on the configuration stored in the memory. As explained before, when the law of motion was computed, a new motion started from the actual position to correct the number of steps that need to be executed to restore the position and avoid an excessive starting speed. The plot also shows that when the external force was removed, the last computed law of motion followed as expected.

**Figure 19:**
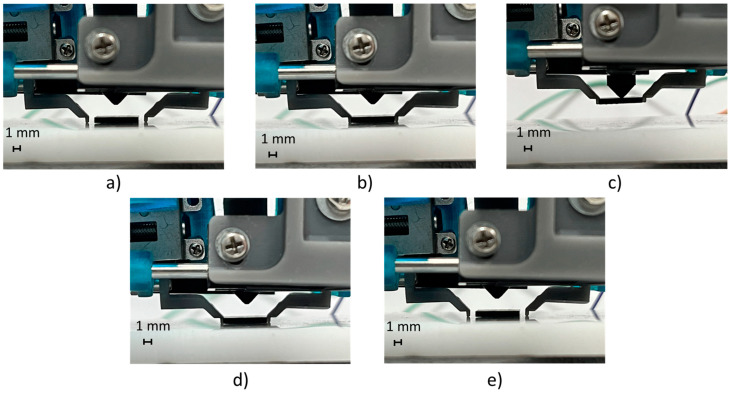
Picking and releasing demonstration exploiting hybrid manipulation: (**a**) initial position; (**b**) picking position of the fingers (mechanical picking); (**c**) upward movement (by the vertical axis of the gripper fingers, i.e., pneumatic cylinder) and vacuum picking; (**d**) downward movement (by the vertical axis of the gripper fingers); and (**e**) releasing position of the fingers.

Finally, the hybrid manipulation mode combining mechanical and vacuum picking was tested by executing 30 cycles of pick-and-release operations. Figure 19 shows the manipulation sequence of the BGA package with dimensions of 8 × 6 mm. The test result exploiting both picking technologies demonstrated that the designed gripper was able to manipulate SMT components in the dimensional range of 0.5–8 mm, with a reliability of 100%.

## 4. Conclusions

The research paper presented the design and development of a multi-mode hybrid micro-gripper aimed at addressing the challenges posed by the increasing complexity and variety of electronic boards based on Surface Mount Technology (SMT). By combining vacuum and friction handling technologies, the proposed micro-gripper offers a versatile solution for manipulating and assembling a wide range of micro- and meso-SMT components with diverse dimensions (from 0.5 mm to 8 mm) and properties. This approach contributes to the ongoing efforts to enhance the reconfigurability and flexibility of workstations in smart manufacturing, handling the high density of components with different sizes and shapes to be assembled on the PCB and minimizing set-up time. The micro-gripper presented in this paper is capable of both open-loop and closed-loop position control of fingers, exploiting sophisticated algorithms for performance improvement and allowing for the achieving of a closed-loop control tolerance of ±50 µm. It is also capable of detecting a possible stall of one or both axes, ensuring that the motor stops within the set threshold to avoid problems of overheating and/or damage to itself or the impacted component. In addition, closed-loop control maintains minimal axis synchronization error over a wide speed range. The control system can perform load disturbance rejection through a designed technique that takes into account the maximum starting frequency of the motors and ensures that the position is restored within the control tolerance.

Higher positioning performance can be achieved with more accurate measurement systems but at a higher cost. The project aimed to meet the specifications required for the assembly of SMT components while maintaining low costs. Future works will consider more accurate and less noisy measurement systems, along with a higher micro-stepping resolution of the driver, in order to achieve better positioning performance.

The development of flexible devices, along with their hardware and software integration and control, is crucial for the future of small batch production and pre-production processes. As the demand for more advanced electronic products continues to grow, researchers must continue to explore and develop innovative solutions that can adapt to the ever-changing landscape of electronic manufacturing. The multi-mode hybrid micro-gripper presented in this paper serves as a promising step toward achieving these high-level objectives and ensuring high manipulation precision and a cost-effective solution for the industrial sectors involved.

The development of the closed-loop control system of the fingers started from the calibration of the linear potentiometers used as position feedback. Their filtered output signal was also analyzed to evaluate the standard deviation of the measurement and was revealed to be suitable for the precision requested for the application. Successively, characterization tests were performed on both axes, both to estimate the mechanical backlash, which resulted to be less than 0.4 mm, and to carry out their identification, to fine-tune the PID controllers for the closed-loop control. Validation trials presented an excellent result with a fit of the estimated data with the real data above 96%. Subsequently, two PI controllers were realized and fine-tuned with a trade-off between disturbance rejection and setpoint following. A feedforward action was also introduced to improve the performance of the setpoint following. Finally, control performance assessment tests were carried out. The first trial consisted of a precise grasping of a sample component, that, thanks to the little control tolerance, was completed. The second trial involved the grasping of the same sample component with the stall detection option activated which was very successful in detecting the impact and properly stopping the motion. During the reopening of the fingers, an obstacle was placed in such a way as to cause a collision with one of the two fingers. Also, in this case, the control system successfully recognized the stall and stopped the movement. The last verification test concerned the rejection of load disturbances, simulated through the application of an external force on the two fingers. The result was excellent, as the control system brought the fingers back into position, within the control threshold, following the desired law of motion.

The micro-gripper respects the project specifications by demonstrating a reliability of 100% in manipulating SMT components in the range of 0.5–8 mm by exploiting vacuum, mechanical, and hybrid picking.

The micro-gripper presented turns out to be highly flexible and scalable, both because of the way it is physically designed and because of software developed, which, thanks to its object-oriented logic, allows its functions to be easily upgraded and new ones to be added. The micro-gripper and its control system can be easily integrated into other systems because of the communication techniques already implemented and because of the possibility of easily adding different ones due to the nature of the system itself.

## Figures and Tables

**Table 1:** Miniature stepper motor technical data.

Name	Value
Type	2-phase 4-wire micro-claw stepper motor
$V_{w}$	3 ÷ 5 V DC
$I_{w,max}$	280 ÷ 300 mA
$R_{w}$	14.5 Ω
Step Angle (full step)	18°
Weight	3.5 g
Gear Reduction Ratio	1/2
Screw shaft pitch	1 mm
Output shaft diameter	1.5 mm
Stroke	9.1 mm
Linear Step (full step)	25 µm

**Table 2:** The standard deviation for the unfiltered potentiometer signal (150,000 samples acquired with a sample time of 0.5 ms).

Position	First Finger	Second Finger
Open	17.67	24.72
Mid-range	14.96	12.48
Close	9.20	9.16

**Table 3:** The standard deviation for the filtered potentiometer signal (150,000 samples acquired with a sample time of 0.5 ms).

Position	First Finger	Second Finger
Open	2.23	2.71
Mid-range	1.72	1.16
Close	0.94	0.91

**Table 4:** Maximum mechanical backlash.

First Finger	Second Finger
388 [µm]	381 [µm]

**Table 5:** First-order model constants for both fingers.

Finger	T	K
1	24.15	853.1
2	73.49	2529.4

**Table 6:** PID controller values for both fingers.

Finger	K_p_	K_i_	K_d_
1	0.1020	0.0064	0
2	0.1223	0.0090	0

## Data Availability

The data presented in this study are available on request from the corresponding author.
